# Phylogenetic and drug- and vaccine-resistance profiles of Hepatitis B Virus among children with HIV co-infection in Pakistan

**DOI:** 10.1016/j.meegid.2022.105371

**Published:** 2022-11

**Authors:** Nida Farooqui, Fatima Mir, Dilsha Siddiqui, Aneeta Hotwani, Apsara Ali Nathwani, Syed Faisal Mahmood, Kamran Sadiq, Hammad Afzal Kayani, Saqib Ali Sheikh, Sharaf Ali Shah, Rashida Abbas Ferrand, Syed Hani Abidi

**Affiliations:** a Department of Biological and Biomedical Sciences, Aga Khan University, Karachi, Pakistan; b Department of Biosciences, The Shaheed Zulfikar Ali Bhutto Institute of Science and Technology, Karachi, Pakistan; c Department of Pediatrics and Child Health, Aga Khan University, Karachi, Pakistan; d Department of Medicine, Aga Khan University, Karachi, Pakistan; e Sindh AIDS Control Program, Ministry of Health, Sindh, Pakistan; f Bridge Consultants Foundation, Karachi, Pakistan; g Department of Clinical Research, London School of Hygiene & Tropical Medicine, London, United Kingdom; h Department of Biomedical Sciences, Nazarbayev University School of Medicine, Nur-Sultan, Kazakhstan

**Keywords:** HIV-1, HBV co-infection, Phylogenetics, Drug resistance, Vaccine escape, Outbreak

## Abstract

**Introduction:**

HIV-1 and hepatitis B virus (HBV) share common routes of transmission and therefore co-infection is common. In 2019, an HIV-1 outbreak that resulted in >1000 children being infected, predominantly through nosocomial transmission, occurred in Sindh, Pakistan. We conducted a phylogenetic and drug resistance analysis of the HBV Reverse Transcriptase (RT) gene in children with HIV-1 and HBV co-infection.

**Methodology:**

Blood samples were collected from 321 children with HIV who were recruited as part of a study to investigate the HIV-1 outbreak. All samples were tested for HBV surface antigen (HBsAg) using an ELISA assay, and positive samples were used to amplify and sequence the HBV RT gene. The phylogenetic relationship between sequences was analyzed, and drug- and vaccine- resistance mutations in the RT gene were explored.

**Results:**

Of 321 samples, 23% (*n* = 75) were positive for HBsAg on ELISA. Phylogenetic analysis of the sequences revealed that 63.5% of HBV sequences were sub-genotype D1, while the rest were sub-genotype D2. Cluster analysis revealed grouping of sub-genotype D1 sequences exclusively with Pakistani sequences, while clustering of sub-genotypes D2 predominantly with global sequences. The 236Y mutation associated with resistance to tenofovir was observed in 2.8% of HBV sequences. Additionally, seven vaccine escape mutations were observed, the most common being 128 V.

**Conclusion:**

Our study suggests ongoing transmission of HBV D1 and D2 sub-genotypes in the HIV-1 co-infected population, likely nosocomially, given common routes of HVB and HIV-1 transmission. The prevalence of major HBV drug- and vaccine-resistant mutations remains low. Surveillance for further transmissions and the possible emergence of major drug- or vaccine-resistant variants is required.

## Highlights

• HBV sub-genotype D1 is the prevalent genotype in Pakistan
• Ongoing transmission of HBV D1/D2 sub-genotypes in the HIV co-infected population, likely through nosocomial routes.
• The prevalence of major HBV drug- and vaccine-resistant mutations remains low.
• Surveillance for further transmissions and the possible emergence of major drug- or vaccine-resistant variants is required.

## 1 Introduction

HIV-1 and hepatitis B virus (HBV) share common routes of transmission and therefore co-infection is common (Sayan et al., 2020). HBV and HIV-1 can be transmitted to children through contaminated blood products or via vertical transmission (Yeung and Roberts, 2001). Untreated HBV infection may lead to chronic hepatitis or even further progress to hepatocellular carcinoma (Schaefer and John, 2021).

HBV-encoded enzyme reverse transcriptase (RT) is vital for several steps of viral replication, such as DNA priming, genome repair, and assembly (Clark and Hu, 2015). All approved antiviral drugs for the treatment of chronic HBV, target the RT region (Clark and Hu, 2015). In both adults and infants infected with HIV-1, HBV treatment includes antiviral therapy (ART) comprising lamivudine and tenofovir disproxil fumarate (TDF), which are active drugs against HBV reverse transcriptase (Schmutz et al., 2006). Mutations in the reverse transcriptase gene of HBV, such as in the YMDD motif, cause resistance against tenofovir and lamivudine leading to the failure of HBV therapy (Yeh et al., 2000).

Pakistan has a high prevalence of HBV infection, which varies between provinces. Several recent studies from the year 2021–22 showed a high prevalence of HBV infection (detected either through a positive PCR or positive HBsAg serum test) in adults varying from 80% in Sindh (Jamali, 2022), 50% in Khyber Pakhtunkhwa (Muhammad et al., 2022), 65% in Punjab (Raza et al., 2022), 17% in Baluchistan (Sheikh et al., 2011) and 28.1% in Azad Jammu and Kashmir (Mahmood et al., 2022). Epidemiological studies have focused predominantly on people who inject drugs and incarcerated individuals. In addition, the molecular epidemiology of HBV is poorly understood, and no studies have investigated HBV drug resistance or phylogenetic profiles.

HIV-1 exists in Pakistan as a concentrated epidemic in key populations, especially among men who have sex with men (MSM) and people who inject drugs (PWID) with a prevalence of around 38–40 and 7.5%, respectively (Hasan et al., 2018; Waheed and Waheed, 2017). However, very little is known about the dynamics of HIV-1 and co-infecting viruses such as HBV, HCV, etc., in the pediatric population of Pakistan (Mir et al., 2020; Raees et al., 2013; Waheed and Waheed, 2017). In 2019, an HIV-1 outbreak occurred among children of Sindh province (Larkana District) of Pakistan, where >1000 children got infected. Prior to the 2019 outbreak, three other HIV outbreaks occurred in Larkana - the first among PWID in 2003, the second in 2016 among 12 children in a pediatric hospital, and the third in 2016 among 56 individuals in a renal dialysis unit, mainly due to poor infection control practices (Altaf et al., 2016; Brenner et al., 2007). The 2019 outbreak, however, was unprecedented as it predominantly involved large numbers of children with no known HIV status or risk factor (Mir et al., 2020; Raees et al., 2013; Waheed and Waheed, 2017). The investigations so far have suggested that HIV-1 infections were most likely nosocomially transmitted (Abidi et al., 2022; Siddiqui et al., 2020). In our recent report, we identified high prevalence of CMV and EBV among these children (Nazim et al., 2022), however, the prevalence and phylodynamics of HBV among these children is unknown. Therefore, in this study, we analyzed the phylogenetic and drug resistance profile of the HBV Reverse Transcriptase (RT) gene in children with HIV-1 with HBV co-infection.

## 2 Methods

### 2.1 Sample collection

Blood samples were previously collected from 321 children diagnosed with HIV-1 during the 2019 outbreak investigation (Abidi et al., 2022). The samples were collected after obtaining written informed assent from participants and informed consent from the parents/guardians. Briefly, demographics and clinical data including age, sex, ethnicity, parent's HIV/HBV status if known, and ART history including duration of therapy (in participants with HIV) was collected through a questionnaire. The study was approved by the Aga Khan University Ethical Review Committee (approval # 1536–4200-AKU-ERC-2019). Approximately 77% of the participants had a median age of three (IQR: 2–5) years. Approximately, 64.79% of the subjects were males and 35% were females. At the time of sample collection, 84% of the children were taking ART for a median of 41 days (IQR 22–192 days), and the regimen included both lamivudine and tenofovir. The serum samples were tested for Hepatitis B surface antigen (HBsAg) test using a commercially available ELISA kit (Enzygnost HBsAg monoclonal, Behring Germany) and following the manufacturer's instructions.

### 2.2 Genomic DNA isolation, quantification, PCR amplification, and sequencing

Viral DNA was extracted from HBsAg-positive blood samples using QIAamp DNA Mini Kit (Hilden, Germany) following the manufacturer's instructions. The extracted DNA was stored at −80 °C until further use. The DNA samples were quantified on NANODROP Spectrophotometer (Thermo Scientific NanoDrop, Catalog number: ND-2000); the samples with an A260/A280 ratio of 1.7–1.8 nm were considered purified DNA. For subsequent steps, samples with a DNA concentration > 50 ng/ul were used.

PCR amplification of the HBV polymerase (reverse transcriptase [RT]) gene was performed using 2× Taqplus Mastermix (ABM). The fragment of the RT gene was amplified using the following primers: F1: 5′-GTGTGGATTCGCACTCCT-3′ and R1: 5′-CGTCAGCAAACACTTGGC-3′ amplifying a region with a fragment length of 2.1 kb. The thermocycling conditions were: initial denaturation at 94 °C for 2 min, 35 cycles of denaturation at 94 °C for 40 s, annealing at 60 °C for 90 s, and extension at 68 °C for 3 min, with a final extension at 68 °C for 8 min (Chavan et al., 2017). The samples failing to amplify with the afore-mentioned primer set were amplified using primer set 2: F2: 5’-CCAGAGTGAGGGGCCTATATT-3′ and R2: 5’-GCGAGCAAAACAAGCTGCTA-3′ amplifying a region with a fragment length of 1270 bp, or primer set 3: F3 (5′- CAAGGTATGTTGCCCGTTTG −3′) and R3: (5’-CCCAACTCCTCCCAGTCCTTAA-3′) amplifying a fragment length of 1250 bp. The thermocycling conditions for primer set 2 were: initial denaturation at 94 °C for 3 min, followed by 35 cycles of denaturation at 94 °C for 30 s, annealing at 56 °C for 50 s, and extension at 72 °C for 2 min, with a final extension at 72 °C for 10 min (Fares and Holmes, 2002), while thermocycling conditions for primer set 3 were: initial denaturation at 94 °C for 5 min, then 30 cycles of denaturation at 94 °C for 45 s, annealing at 55 °C for 30 s and extension at 72 °C for 30 s, with a final extension at 72 °C for 10 min (Archampong et al., 2017). The amplified products were sequenced from Eurofins Scientific, and sequences were deposited to the NCBI GenBank and were assigned accession numbers MZ450809-MZ450867.

### 2.3 Determination of HBV genotype and phylogenetic analysis

The amplified HBV pol sequences were edited and cleaned using MEGA 7 software (Kumar et al., 2016) and given a unique ID (starting with AKULO). The initial genotyping for each sequence was performed using the geno2pheno tool (Beggel et al., 2009). The genotypes were further confirmed using phylogenetic analysis. For phylogeny, HBV genotype A, B, C, D1, D2, and E RT sequences were used as reference datasets (Supplementary File 1). Using the MAFFT alignment tool (Edgar and Batzoglou, 2006) HBV sequences from our participants were aligned with the above-mentioned reference sequences. The aligned sequences were subsequently used to construct the Maximum Likelihood (ML) tree using the IQTREE tool, with the following parameters: substitution model: Gamma-distribution (GTR) with invariant sites (G + I), and single branch tests with 1000 replicates of Shimodaira Hasegawa (SH-aLRT) (Trifinopoulos et al., 2016). Nodes with an aLRT support value of >90% were considered significant.

### 2.4 Cluster analysis

For cluster analysis, three sequence sets were used, HBV sequence obtained from our participants, global reference sequences (matching each D1 or D2 sequence on BLAST search), and Pakistani D1 and D2 reference sequences, retrieved separately from NCBI (Supplementary File 2). The D1 and D2 sequence sets were aligned separately as described above. These aligned sequences were then used to construct the ML tree using the IQTREE tree software, using the parameters described above.

### 2.5 Effective population size and the time to the most recent common ancestor (tMRCA)

The time to the most common recent ancestor (tMRCA) and effective population size of HBV subtype-genotype (D1 and D2) clusters were estimated using a Bayesian Markov Chain Monte Carlo (MCMC) analysis in BEAST software v1.10.4 (Suchard et al., 2018). The study sequences (*n* = 55 D1 and *n* = 14 D2) were amplified from samples in 2019 and did not independently have a sufficient temporal signal for inference of the dates of origin. Hence, additional Pakistani reference sequences (*n* = 147 D1 and *n* = 17 D2) (Supplementary File 2) sampled from different years (1995–2019) on BLAST search matching our study sequences were combined to inform the temporal signal (Katoh and Standley, 2013). Sub-genotype Bayesian inferences were performed in BEAST 1.10.4 using the Bayesian Skyline model with an uncorrelated lognormal relaxed clock and inferred under the GTR + Γ4 + I substitution model (Suchard et al., 2018). BEAST runs of 200 million (for D1) and 100 million (for D2) generations were performed, sampling every 20,000th and 10,000th iteration, respectively, and discarding the first 10% of samples as burn-in. The run gave an effective sample size (ESS) ≥200, which was analyzed using Tracer v.1.7.0 software (Suchard et al., 2018).

### 2.6 Analysis of drug resistance and vaccine escape mutations

Each study sequence was analyzed for the presence of drug resistance mutations in the RT region using the geno2pheno tool (Beggel et al., 2009). Canonical and non-canonical mutations, associated with resistance to lamiv\udine and TDF were analyzed (Döring et al., 2018). Since the polymerase region of HBV overlaps with Hepatitis B surface antigen (HBsAg) (Ding et al., 2016), we also used this region to detect the presence of vaccine escape mutations using the geno2pheno tool.

## 3 Results

### 3.1 Genotype analysis of HBV sequences

Of 321 samples, 23% (*n* = 75) were positive for HBsAg. Of 75 HBsAg-positive samples, 71 samples were amplified on PCR. Two samples were excluded because of the presence of multiple stop codons, leaving 69 samples for analysis. Initial genotyping revealed *n* = 55 and *n* = 14 sequences to be HBV sub-genotypes D1 and D2 respectively.

**Fig. 1:**
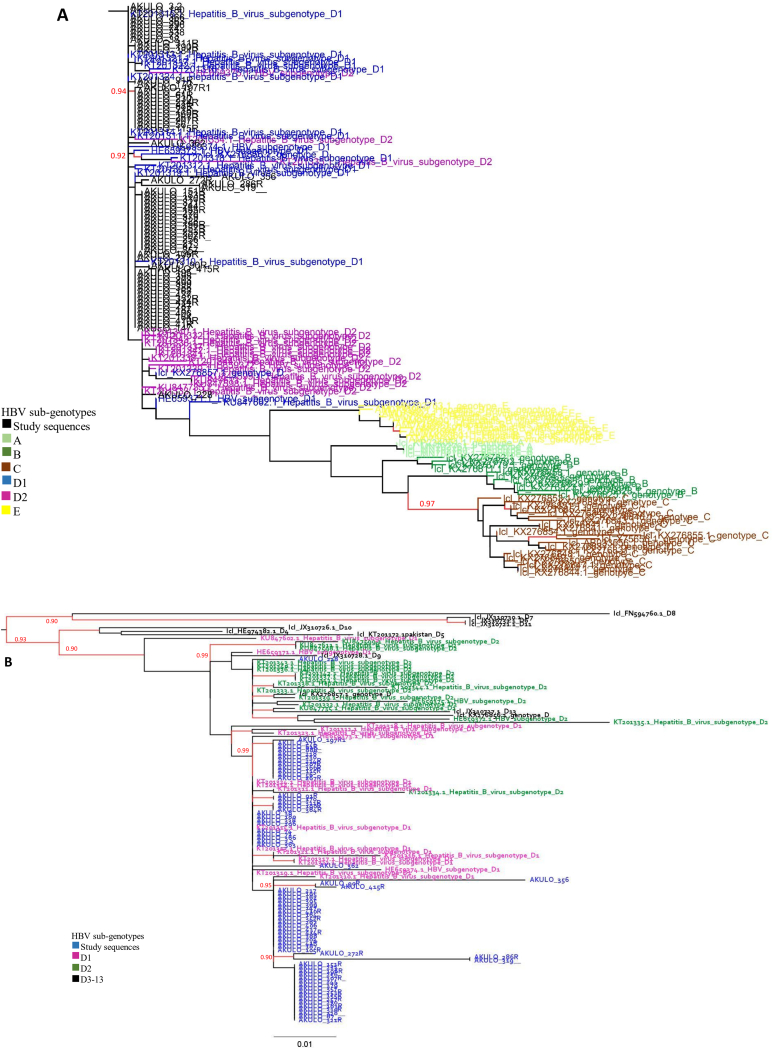
A. Genotypic analysis of HBV pol sequences: Maximum likelihood-based subtype analysis of HBV pol (*n* = 154) sequences. The color codes of the nodes and branches are described in the legend. The tree scale represents the nucleotide per substitution site. Nodes with significant (≥90) aLRT values are shown in red color. Fig. 1B Sub-genotypic analysis of HBV pol sequences: Maximum likelihood-based subtype analysis of HBV sub-genotype pol (n = 154) sequences. The color codes of the nodes and branches are described in the legend. The tree scale represents the nucleotide per substitution site. Nodes with significant (≥90) aLRT values are shown in red color. (For interpretation of the references to color in this figure legend, the reader is referred to the web version of this article.)

This observation was further confirmed using the phylogenetic analysis (Fig. 1A), whereas no sequences matched sub-genotype A, B, C, and E (Fig. 1A).

### 3.2 Cluster analysis and estimation of tMRCA for D1 and D2 sub-genotype clusters

**Fig. 2:**
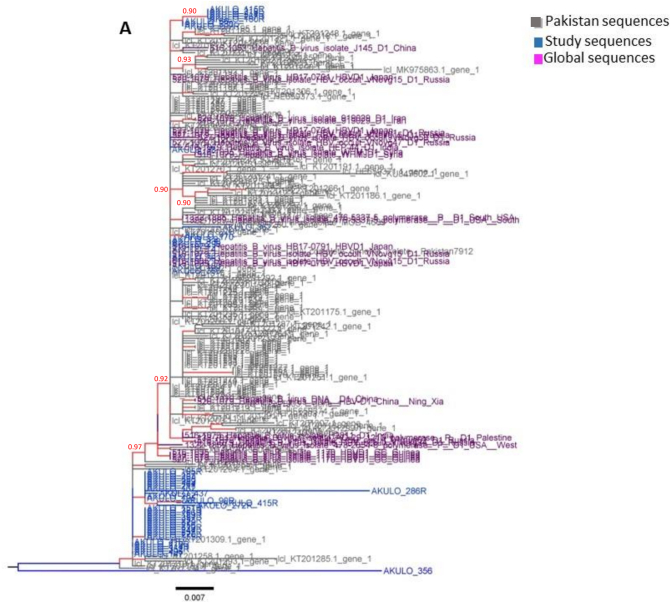

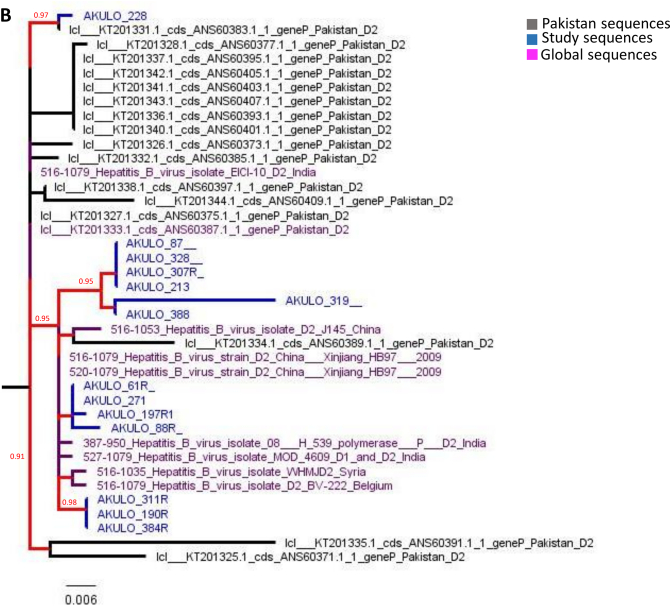
HBV Cluster analysis: Trees represent the Maximum Likelihood tree-based cluster analysis for A) 233 HBV sub-genotype D1 and B) 39 HBV sub-genotype D2 *pol* sequences comprising the study sequences as well as Pakistani, and global reference sequences. The branches and nodes are color-coded according to the legend. The cluster tree is rooted at the midpoint with increasing node order. The nodes with significant (≥90) SH-aLRT values are shown in light grey color. The tree scale represents the nucleotide per substitution site.

The cluster analysis showed the D1 genotype sequences from our study to form multiple clusters predominantly with the Pakistani D1 genotype reference dataset, indicating local HBV sub-genotype D1 transmission (Fig. 2A). The D2 genotype sequences clustered with sequences from India, China, Syria, and Belgium (Fig. 2B), indicating multiple introductions of sub-genotype D2 in Larkana followed by local transmission.

### 3.3 Effective population size and the time to the most recent common ancestor (tMRCA)

**Fig. 3:**
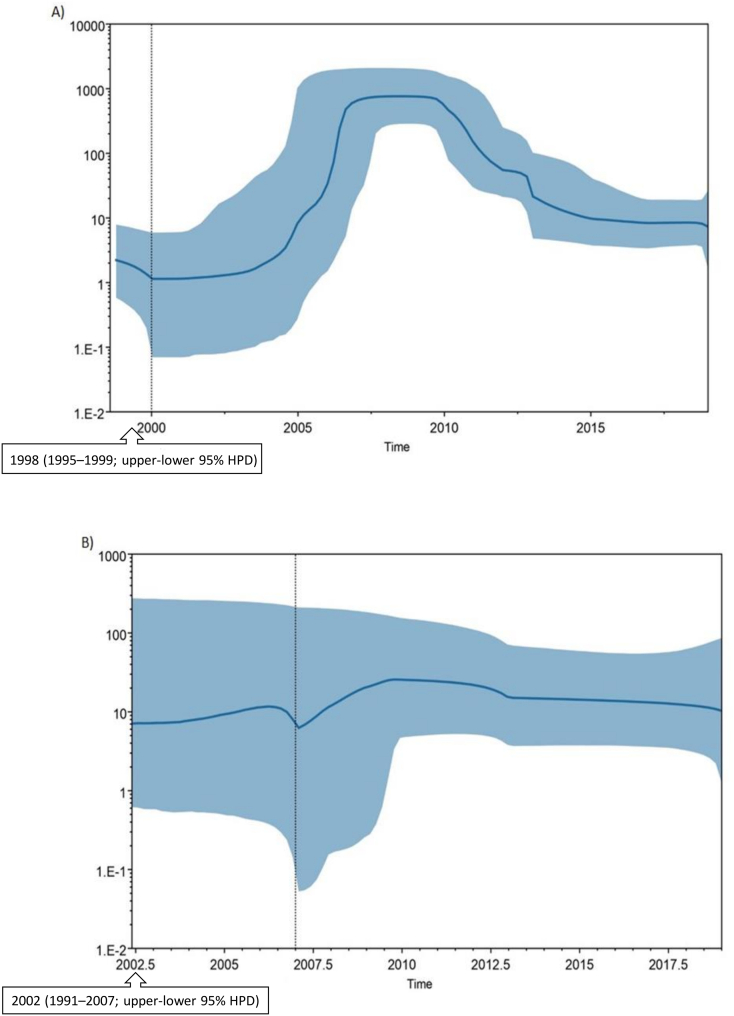
HBV D1 and D2 sequences effective population size and time to the most recent common ancestor. A Bayesian Skyline plot, based on a ‘relaxed clock’ coalescent framework analysis, was constructed using a combined (A) Pakistani reference and D1 study sequences (*n* = 147 and *n* = 55), and (B) Pakistani reference and D2 study sequences (*n* = 17 and n = 14) representing all years (1995–2019), respectively. The X-axis represents time in years, while Y-axis shows the effective population size. The thick blue line represents the median, while the blue band represents 95% highest posterior density (HPD) intervals. The tMRCA of HBV sub-genotypes D1 and D2 is indicated by a black arrow box. (For interpretation of the references to color in this figure legend, the reader is referred to the web version of this article.)

The time to the most recent common ancestor (tMRCA) was estimated to be around 1998 (1995–1999; upper-lower 95% HPD) for HBV sub-genotype D1 (Fig. 3A, black-boxed arrows) and 2002 (1991–2007; upper-lower 95% HPD) HBV sub-genotype D2 (Fig. 3B, black-boxed arrows). The evolutionary rates for HBV sub-genotypes D1 and D2 were estimated to be 1.5 × 10^−3^ and 2.3 × 10^−3^ substitutions site per year, respectively. Moreover, we further estimated the past population dynamics of HBV sub-genotype D1 and D2 using Bayesian skyline plot (BSP) analysis, which showed an estimated expansion in population size of the sub-genotype D1 between the years 2012 to 2018 (Fig. 3A), whereas, for sub-genotype D2, the effective population size remained uniform throughout the years (Fig. 3B).

### 3.4 Drug resistance and vaccine escape mutations

Mutations 236D, 236H, 236Y, and 184I mutations were found in 2.8% (*n* = 2) of the study sequences and were associated with resistance to adefovir, TDF, and entecavir (Yano et al., 2015). Mutations 184 M, 184S, and 184 N were found in one participant and were associated with resistance to entecavir (Yano et al., 2015).

In addition to the canonical drug resistance mutations, several other unclassified mutations were also found in the HBV study sequences (Supplementary Table 3). Among these, the most common were RT-N248H and RT-Y135S, which were observed in 97% (*n* = 69) and 84% (*n* = 60) of the study sequences (Supplementary Table 3). These mutations are not associated with any drug resistance or vaccine escape and may most likely be viral polymorphisms.

Analysis of the vaccine-escape mutation showed vaccine-escape mutation 137R in 7% (*n* = 5) of the study sequences. Similarly, two 2.8% (2.8%) study sequences harbored secondary (resistances/mutations outside of the active site of the protein (Clemente et al., 2003)) mutation 128 V, while one participant harbored mutations 129R, 137Y, 144E, 124R, and 141E (Adesina et al., 2021).

We also analyzed drug- and vaccine-resistance mutations in Pakistani reference sequences matching our study sequences. None of the Pakistan reference sequences exhibited any major drug resistance mutations. However, similar to our study sequences, non-canonical mutations RT-N248H and RT-Y135S were observed in 91% (*n* = 151) and 90% (*n* = 150) of Pakistani reference sequences (Supplementary Table 4). Analysis of vaccine-escape mutations showed the presence of mutations 143 L in 6.7% (*n* = 11), and D144A, 145A, and 145R in 1.2% (n = 2) of the Pakistani reference sequences.

## 4 Discussion

Our study is the first to report a phylodynamic and mutational analysis of Pakistani HBV sub-genotype D1 and D2 sequences. In our study, 23% of the children with HIV-1, recruited from the cases of the Larkana 2019 outbreak, were found positive for HBV sub-genotypes D1 and D2.

HBV comprises of ten genotypes A-J (Sunbul, 2014), with variable distribution worldwide (Bollyky et al., 1996; Fares and Holmes, 2002; Norder et al., 1992; Stuyver et al., 2000). HBV sub-genotype D1 is most prevalent in Pakistan (Hanif et al., 2013). In our study, D1 was found to be the predominant sub-genotype, sharing a close phylogenetic relationship with Pakistani reference sequences, and yielded a combined tMRCA of 1998 (1995–1999; upper-lower 95% HPD) as seen in our Bayesian analysis, suggesting domestic acquisition and transmission of sub-genotype D1 within Larkana. In contrast, HBV sub-genotype D2, found to be the minor HBV sub-genotype in our cohort, showed linkages with sequences from India, China, Syria, and Belgium, suggesting the introduction of HBV in Larkana around 2002 (1991–2007; upper-lower 95% HPD), likely followed by domestic transmission of D2 sub-genotype in Larkana.

Our previous analysis of HIV-1 sequences amplified from these samples suggested HIV-1 epidemiological transmission in Larkana to occur through nosocomial routes but since the infected infants were coming from various hospitals, the source of introduction of the infection could be multiple (Altaf et al., 2019; Rabold, 2021), and since these samples are co-infected with HBV, it is safe to assume that HBV transmission also occurred through this route. The possibility of vertical transmission of HBV in this cohort also exists, however, this could not be confirmed due to the unavailability of maternal samples. Furthermore, prior to this outbreak, the HIV/HBV status of these children was not known, hence, nosocomial acquisition of HBV appears to be the most plausible route of transmission in these children. High rates of HBV co-infection, mainly acquired through nosocomial transmission, have also been previously reported among individuals with HIV-1 in Europe, the USA, Australia, and Saudi Arabia (Alter, 2006; Bartholomeusz et al., 2004; El-Hazmi, 2004; Lincoln et al., 2003) and also among children with HIV-1 (Lawal et al., 2020; Yendewa et al., 2021).

The treatment of choice against HBV comprises reverse transcriptase inhibitors: tenofovir (Tallo et al., 2008) and lamivudine (Allen et al., 1998). These drugs restrict the reverse transcription process near the YMDD motif (Kim et al., 2010). In our dataset, no major mutations conferring resistance against tenofovir, and lamivudine were observed which shows that the HBV strains in Pakistan are still susceptible. In addition to drug resistance mutations, 7% of the study participants harbored mutation 137R, associated with reduced vaccine efficacy against HBV (Mirandola et al., 2011). This suggests that the available vaccine and drugs are effective against HBV strains circulating in Pakistan. This is further supported by our Bayesian analysis which showed a steady decline in HBV effective population size after 2011, which could be due to effective vaccine coverage or resolution of HBV infection around 2012. The diminishing cases of HBV observed after 2019 may be attributed to the national HBV immunization programs (Extended Program on Immunization (EPI) initiated in 2009) that have successfully vaccinated >160,000 individuals in Larkana between 2009 and 2022 (personal communication with the EPI-Sindh officials) that have had a significant impact in reducing the HBV infections (Rabold, 2021). Additionally, in absence of studies/reports on COVID-related lockdowns on the decrease in transmission of other communicable diseases in Larkana, the efficacy of lockdown of decrease in HBV cases after 2019 cannot be ascertained.

Limitations include the absence of maternal samples, limiting the ability to identify vertical transmission of HBV. Secondly, the sample size to establish HBV transmission networks was low. Lastly, the sample size was collected from children visiting a single or limited number of healthcare facilities, hence, there may be bias in the result of this estimate.

## Significance

This study is the first to report phylodynamic and mutational analysis of HBV in the HIV-1 pediatric cohort of Pakistan. The study suggests continuous ongoing transmission of HBV, along with HIV-1 in Larkana (Abidi et al., 2022). There is a need for molecular surveillance for monitoring transmissions, and the possible emergence of major drug- or vaccine-resistant variants.

## Author contributions

“Conceptualization, SHA; methodology, NF, FM, DS, AH, HAK; formal analysis, NF, AH, DS; outbreak investigation, AAN, FM, SFM, KS, SS, SAS, RAF, SHA; writing—original draft preparation, NF, FM, DS; writing—review and editing, RFA, SHA; supervision and funding acquisition, RFA, SHA. All authors have read and agreed to the published version of the manuscript.”

## Declaration of Competing Interest

The authors declared no competing conflict of interest.

## Data Availability

The data is given in the supplementary files

## Appendix A Supplementary data

Supplementary material 1 Image 1

Supplementary material 2 Image 2

Supplementary material 3 Image 3

Supplementary material 4 Image 4
