# Supplementary material for: Phylogenetic and drug- and vaccine-resistance profiles of Hepatitis B Virus among children with HIV co-infection in Pakistan
Source: Infect Genet Evol. 2022 Nov;105:105371. doi: 10.1016/j.meegid.2022.105371 (PMC9614405; doi:10.1016/j.meegid.2022.105371)
Supplement: Supplementary file 1 — Supplementary material 1 [file mmc1.docx]

**Supplementary File 1:**

The reference sequences included in the study can be retrieved using following accession numbers,

**Sub-genotype A:** KP234051.1, MN702760.1, MN702761.1, MN702762.1

**Sub-genotype B:** KX276858.1, KX276829.1, KX276828.1, KX276825.1, KX276823.1, KX276792.1, KX276782.1, KX276826.1, KX276779.1, KX276824.1, KX276811.1

**Sub-genotype C:** KX276855.1, KX276854.1, KX276853.1, KX276852.1, X75656.1, KX276851.1, KX276850.1, KX276849.1, KX276848.1, KX276847.1, KX276846.1, KX276845.1, KX276844.1, KX276844.1, KX276843.1, KX276842.1, KX276841.1, KX276841, KX276837.1, KX276831.1, AB033556.1

**Sub-genotype D1:** KU847602.1, HE659374.1, KT201324.1, KT201313.1, HE659371.1, KT201321.1, KT201312.1, KT201319.1, KT201314.1, KT201310.1, KT201315.1, KT201317.1, KT201316.1, KT201323.1, KT201311.1, KT201322.1, KT201318.1, HE659373.1, KX276856.1, KX276857.1;

**Sub-genotype D2:** KU847611.1, KU847598.1, KU847599.1, HE659372.1, KU847735.1, HE659375.1, KT201335.1, KT201343.1, KT201342.1, KT201337.1, KT201341.1, KT201336.1, KT201338.1, KT201339.1, KT201340.1, KT201333.1, KT201332.1, KT201344.1, KT201334.1; **Genotype E:** AM073602.1, AM073595.1, AM073601.1, AM073598.1, AM073600.1, AM073599.1, AM073597.1, AM073594.1, AM073596.1, MN702763.1;
