# Supplementary material for: Phylogenetic and drug- and vaccine-resistance profiles of Hepatitis B Virus among children with HIV co-infection in Pakistan
Source: Infect Genet Evol. 2022 Nov;105:105371. doi: 10.1016/j.meegid.2022.105371 (PMC9614405; doi:10.1016/j.meegid.2022.105371)
Supplement: Supplementary file 2 — Supplementary material 2 [file mmc2.docx]

**Supplementary File 2:**

**Pakistan sub-genotype D1:** AB583680.1, HE659374.1, HE659373.1, HE659371.1, KU847602.1, KT201292.1, KT201234.1, KT201239.1, KT201240.1, KT201241.1, KT201324.1, KT201323.1, KT201301.1, KT201315.1, KT201272.1, KT201180.1, KT201281.1, KT201273.1, KT201195.1, KT201189.1, KT201190.1, KT201193.1, KT201192.1, KT201313.1, KT201286.1, KT201183.1, KT201314.1, KT201280.1, KT201259.1, KT201310.1, KT201277.1, KT201267.1, KT201263.1, KT201276.1, KT201311.1, KT201284.1, KT201282.1, KT201275.1, KT201274.1, KT201266.1, KT201194.1, KT201257.1, KT201270.1, KT201279.1, KT201304.1, KT201309.1, KT201262.1, KT201187.1, KT201319.1, KT201312.1, KT201184.1, KT201269.1, KT201268.1, KT201176.1, KT201182.1, KT201178.1, KT201317.1, KT201316.1, KT201305.1, KT201306.1 KT201303.1, KT201255.1, KT201256.1, KT201321.1, KT201265.1, KT201297.1, KT201296.1, KT201291.1, KT201254.1, KT201253.1, KT201219.1, KT201235.1, KT201220.1, KT201217.1, KT201214.1, KT201208.1, KT201215.1, KT201202.1, KT201210.1, KT201209.1, KT201201.1, KT201203.1, KT201207.1, KT201206.1, KT201205.1, KT201204.1, KT201226.1, KT201232.1, KT201243.1, KT201200.1, KT201198.1, KT201197.1, KT201199.1, KT201216.1, KT201213.1, KT201231.1, KT201218.1, KT201222.1, KT201212.1, KT201211.1, KT201238.1, KT201244.1, KT201228.1, KT201236.1, KT201230.1, KT201233.1, KT201246.1, KT201250.1, KT201249.1, KT201237.1, KT201174.1, KT201287.1, KT201252.1, KT201225.1, KT201245.1, KT201227.1, KT201247.1, |KT201229.1, KT201242.1, KT201248.1, KT201294.1, KT201293.1, KT201295.1, KT201185.1, KT201307.1, KT201299.1, KT201289.1, KT201290.1, KT201173.1, KT201298.1, KT201300.1, KT201322.1, KT201181.1, KT201261.1, KT201191.1, KT201308.1, KT201302.1, KT201251.1, KT201258.1, KT201188.1, KT201175.1, KT201186.1, KT201318.1, KT201288.1, KT201285.1, JF827280.1, MK975863.1 ;

**Pakistan sub-genotype D2:** KT201335.1, KT201325.1, KT201326.1, KT201332.1, KT201333.1, KT201328.1, KT201337.1, KT201342.1, KT201341.1, KT201343.1, KT201336.1, KT201338.1, KT201340.1, KT201331.1, KT201327.1, KT201344.1, KT201334.1;

**Global sub-genotype D1:** KP322599.1, MK507911.1, GQ183480.1, MN507838.1, LC365689.1, AF280817.1, GQ377589.1, JN257180.1, MK598649.1, MK598644.1, MK598636.1, MK598639.1, MK598643.1, MK598642.1, KC528638.1, JN604206.1, JN604263.1, JN040781.1;

**Global sub-genotype D2:** GU798982.1, KF679994.1, GQ183480.1, HQ833467.1, GQ377589.1, JN257180.1, GU563549.1.
