# Supplementary material for: Phylogenetic and drug- and vaccine-resistance profiles of Hepatitis B Virus among children with HIV co-infection in Pakistan
Source: Infect Genet Evol. 2022 Nov;105:105371. doi: 10.1016/j.meegid.2022.105371 (PMC9614405; doi:10.1016/j.meegid.2022.105371)
Supplement: Supplementary file 3 — Supplementary material 3 [file mmc3.docx]

**Supplementary File 3.** Non-canonical/ other mutations of HBV in AKULO outbreak sequences:

| **Other Mutations** | **Number of patients % (n=71)** |  |
| --- | --- | --- |
| Y135S | 60 (84.5) |  |
| N248H | 69 (97.18) |  |
| L145 | 6 (8.45) |  |
| P281L | 3 (4.2) |  |
| L308 | 3 (4.2) |  |
| F300n.d. | 3 (4.2) |  |
| F300I | 2 (2.81) |  |
| N236DHNY | 2 (2.81) |  |
| I269L | 2 (2.81) |  |
| F273I | 2 (2.81) |  |
| L276C | 2 (2.81) |  |
| P277L | 2 (2.81) |  |
| R280D | 2 (2.81) |  |
| P281C | 2 (2.81) |  |
| I282E | 2 (2.81) |  |
| D283E | 2 (2.81) |  |
| L209S | 2 (2.81) |  |
| L217N | 2 (2.81) |  |
| L228I | 2 (2.81) |  |
| L231S | 2 (2.81) |  |
| L235F | 2 (2.81) |  |
| H133DHNY | 2 (2.81) |  |
| I290T | 2 (2.81) |  |
| L293H | 2 (2.81) |  |
| L294FL | 2 (2.81) |  |
| C303R | 2 (2.81) |  |
| G304D | | 2 (2.81) |
| Y305P | | 2 (2.81) |
| A307V | | 2 (2.81) |
| P310C | | 2 (2.81) |
| L311G | | 2 (2.81) |
| Y312F | | 2 (2.81) |
| C314L | | 2 (2.81) |
| Q316S | | 2 (2.81) |
| T240 | | 2 (2.81) |
| K268N | | 2 (2.81) |
| K270E | | 2 (2.81) |
| L144H | | 2 (2.81) |
| K275D | | 2 (2.81) |
| P310A | | 1 (1.4) |
| F178FILV | | 1 (1.4) |
| L179FILV | | 1 (1.4) |
| L294V | | 1 (1.4) |
| 239IRT | | 1 (1.4) |
| F296V | | 1 (1.4) |
| C303G | | 1 (1.4) |
| P310H | | 1 (1.4) |
| N238T | | 1 (1.4) |
| Q267P | | 1 (1.4) |
| K268N | | 1 (1.4) |
| K270E | | 1 (1.4) |
| R274K | | 1 (1.4) |
| K275D | | 1 (1.4) |
| K285G | | 1 (1.4) |
| L132R | | 1 (1.4) |
| C136*CW | | 1 (1.4) |
| S137*Y | | 1 (1.4) |
| H156Q | | 1 (1.4) |
| I163V | | 1 (1.4) |
| S176IK | | 1 (1.4) |
| L147V | | 1 (1.4) |
| R192S | | 1 (1.4) |
| 302H | | 1 (1.4) |
| I163M | | 1 (1.4) |
| P170R | | 1 (1.4) |
| T184IM | | 1 (1.4) |
| T240K | | 1 (1.4) |
| W284R | | 1 (1.4) |
| T184INST | | 1 (1.4) |
| P277APST | | 1 (1.4) |
| R274 | | 1 (1.4) |
| L146A | | 1 (1.4) |
| S176N | | 1 (1.4) |
| I282HLPR | | 1 (1.4) |
| D283D | | 1 (1.4) |
| 284GR | | 1 (1.4) |
| C287CGRS | | 1 (1.4) |
| R289A | | 1 (1.4) |
| I290I | | 1 (1.4) |
| V291 | | 1 (1.4) |
| L132L | | 1 (1.4) |
| D134D | | 1 (1.4) |
| Q149R | | 1 (1.4) |
