# Supplementary material for: Phylogenetic and drug- and vaccine-resistance profiles of Hepatitis B Virus among children with HIV co-infection in Pakistan
Source: Infect Genet Evol. 2022 Nov;105:105371. doi: 10.1016/j.meegid.2022.105371 (PMC9614405; doi:10.1016/j.meegid.2022.105371)
Supplement: Supplementary file 4 — Supplementary material 4 [file mmc4.docx]

**Supplementary Table 4. HBV vaccine escape mutations in Pakistani reference sequences**.

| **Mutations** | **Reported Impact** | **Mode of action** | **Number of reference sequence n=164 (%)** |
| --- | --- | --- | --- |
| 143L | High | Vaccine, Detection | 11 (6.7) |
| D144A | High | Vaccine, Detection | 2 (1.2) |
| 145A | High | Vaccine | 2 (1.2) |
| 145R | High | Vaccine | 2 (1.2) |
| 126I | Low | Vaccine, Detection | 1 (0.6) |
| 129H | Low | Vaccine | 1 (0.6) |
| 144E | Low | Vaccine, Detection | 1 (0.6) |
| T131I | Low | Vaccine, Detection | 1 (0.6) |
| T126ST | Low | Vaccine, Detection | 1 (0.6) |
| M133I | Low | Vaccine, Detection | 1 (0.6) |
| 134N | Low | Vaccine, Detection | 1 (0.6) |

**Supplementary Table 3. Non-canonical/ other mutations in Pakistani reference sequences**.

| **Other Mutations** | **Number of reference sequence n=164 (%)** |
| --- | --- |
| Y135S | 150 (90) |
| N248H | 151 (92) |
| I266 | 24 (14.6) |
| A223 | 18 (10.9) |
| Q215 | 16 (9.7) |
| E271 | 16 (9.7) |
| C256 | 15 (9.1) |
| N238 | 14 (8.5) |
| Q149 | 11 (6.7) |
| D263 | 10 (6.1) |
| S213 | 8 (4.8) |
| S219 | 8 (4.8) |
| F221 | 5 (3.0) |
| Q267 | 5 (3.0) |
| Y257 | 4 (2.4) |
| I282 | 4 (2.4) |
| N131 | 4 (2.3) |
| I187 | 3 (1.8) |
| V253 | 3 (1.8) |
| I233 | 3 (1.8) |
| L217 | 2 (1.2) |
| R153 | 2 (1.2) |
| V142 | 2 (1.2) |
| I269 | 2 (1.2) |
| M204 | 2 (1.2) |
| D134 | 2 (1.2) |
| I163 | 2 (1.2) |
| R289 | 2 (1.2) |
| V278 | 2 (1.2) |
| K270 | 2 (1.2) |
| I290 | 2 (1.2) |
| R192 | 1 (0.6) |
| Y245 | 1 (0.6) |
| H124 | 1 (0.6) |
| R193 | 1 (0.6) |
| R138 | 1 (0.6) |
| L175 | 1 (0.6) |
| R274 | 1 (0.6) |
| L220 | 1 (0.6) |
| N139 | 1 (0.6) |
| A223 | 1 (0.6) |
| K212 | 1 (0.6) |
| V214 | 1 (0.6) |
| S185 | 1 (0.6) |
| L199 | 1 (0.6) |
| V173 | 1 (0.6) |
| L231 | 1 (0.6) |
| L276 | 1 (0.6) |
| K285 | 1 (0.6) |
| L157 | 1 (0.6) |
| A194 | 1 (0.6) |
| C198 | 1 (0.6) |
| T225 | 1 (0.6) |
| L229 | 1 (0.6) |
| N279 | 1 (0.6) |
| L140 | 1 (0.6) |
| P237 | 1 (0.6) |
| L180 | 1 (0.6) |
| V207 | 1 (0.6) |
| M309 | 1 (0.6) |
